# COVID-19 Delta Variant-of-Concern: A Real Concern for Pregnant Women With Gestational Diabetes Mellitus

**DOI:** 10.3389/fendo.2021.778911

**Published:** 2021-11-15

**Authors:** Md Mahfuz Al Mamun, Muhammad Riaz Khan

**Affiliations:** Translational Research Institute, Henan Provincial People’s Hospital, Henan Key Laboratory of Stem Cell Differentiation and Modification, School of Clinical Medicine, Henan University, Zhengzhou, China

**Keywords:** gestational diabetes mellitus, COVID-19, SARS-CoV-2, Delta VOC, pregnant woman

## Abstract

The emergence of the COVID-19 Delta variant-of-concern (VOC), a novel variant of SARS-CoV-2, has threatened the total health systems throughout the world. This highly contagious strain is spreading at a higher exponential rate than any other variants of COVID-19 by infecting and subsequently killing hundreds of thousands of people globally. Among the most sensitive groups, pregnant women are at high risk of increased hospitalization, pneumonia, respiratory support, and admission to intensive care units during the Delta period. Pregnant people with gestational diabetes mellitus (GDM) are at increased chances of Delta VOC infection. GDM patients are nine and three times more likely to be infected by Delta VOC than those pregnant patients suffering from diabetes and cardiovascular diseases and hypertension, respectively. Additionally, they are more vulnerable to Delta VOC infection than wild-type and Alpha COVID-19 VOC ones. Thus, this review critically sheds light on the current scenario of the vulnerability of pregnant mothers, especially those with GDM, to Delta VOC infection.

## Introduction

The world economy and healthcare systems have collapsed due to the ongoing COVID-19 pandemic (1). The adverse effects of the pandemic on pregnancy have been reported to affect maternal and perinatal health, resulting in higher morbidity and mortality (2–7). Additionally, the emergence of the new SARS-CoV-2 Delta strain, regarded as a variant-of-concern (VOC), has worsened the wellbeing of pregnant women and their babies (8). Pregnant people with comorbidities of diabetes, cardiovascular diseases, and hypertension are vulnerable to COVID-19 infections as much as twice (9). Among the most common pregnancy complications is gestational diabetes mellitus (GDM), defined as an increase in blood sugar (glucose) level during pregnancy due to insulin resistance or insufficient insulin production. This is associated with poor maternal health (depression, preeclampsia, and high risk of cesarean section) and neonatal outcomes (large gestational age, hypoglycemia, high risk of macrosomia, and type 2 diabetes mellitus at adulthood) (10). Here, we have critically reviewed the susceptibility of pregnant women with GDM to Delta VOC infection to inform pregnant mothers, their caretakers, and policymakers to minimize associations of medical complications during pregnancy. In addition, this timely mini review will be a resource providing information on healthcare for the layperson to understand the sensitive complications of SARS-CoV-2 infections during pregnancy.

## Review Protocol

### Search Strategy

PubMed, Scopus, and Google Scholar were the sources of literature searching up to December 2019–September 2021, whereas the Centers for Disease Control and Prevention (CDC), European Centre for Disease Prevention and Control (ECDC), and the WHO provided up-to-date data, statistics, and health-related information about COVID-19. Search terms included COVID-19 or SARS-CoV-2 or 2019-nCoV or novel coronavirus 2019, COVID-19 variant-of-concern, Delta VOC, COVID-19 Delta VOC, COVID-19 variants, gestational diabetes, and evolutionary genomics.

### Selection Criteria

The references of selected original articles, reviews, perspectives, meta-analyses, reports, and guidelines were manually searched. For the data mining and literature review, only those articles, published reports, statistics, or preprints that had been subjected to only COVID-19 and gestational diabetes were taken into consideration. All the authors enlisted herein mutually agreed upon the handpicked articles or reports for analysis during the preparation of the manuscript. Prominence was given to contemplation of information for the average person as well as for general medical readers.

## SARS-CoV-2 Delta Variant-of-Concern and Gestational Diabetes Mellitus

**Figure 1:**
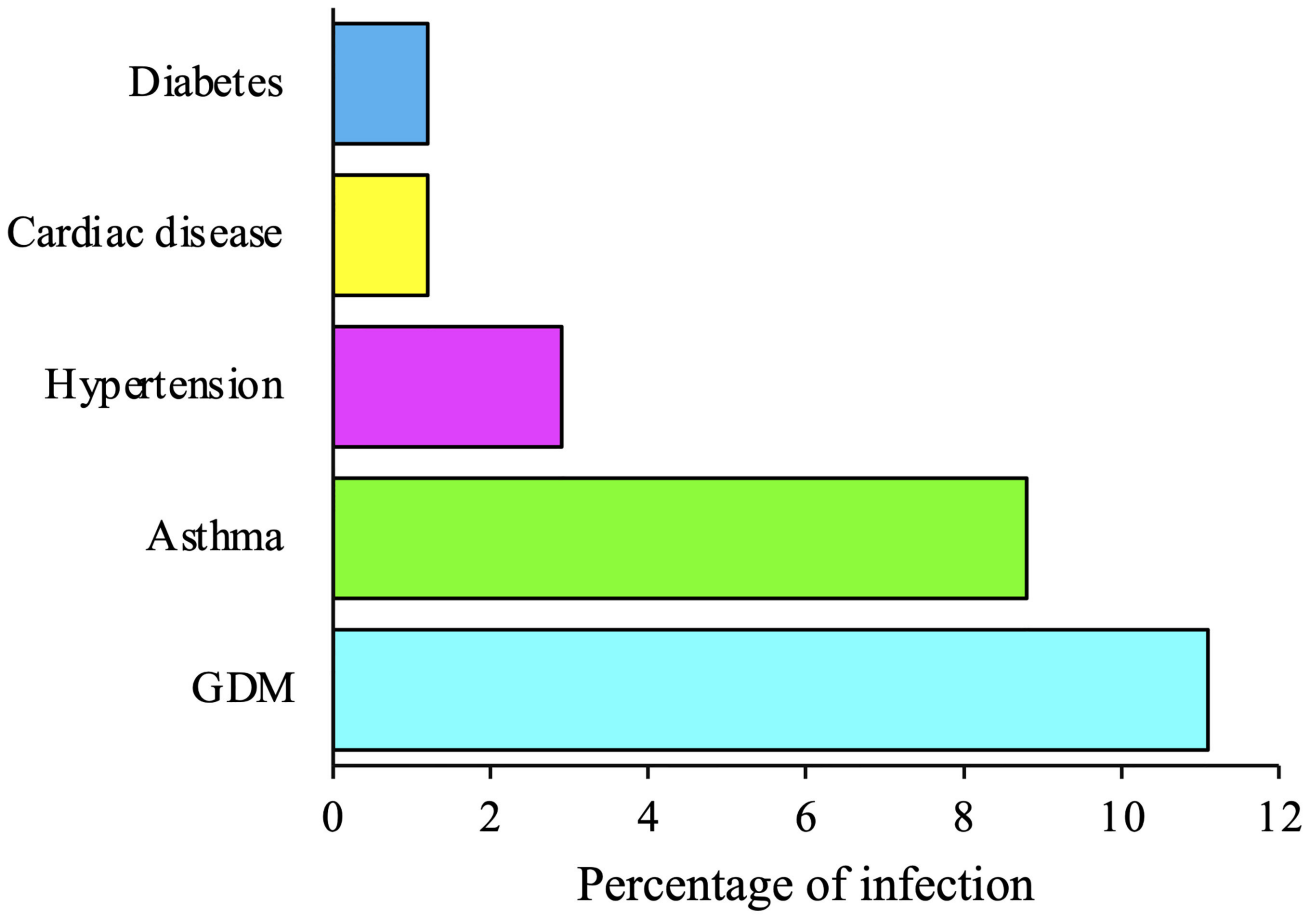
COVID-19 infection of pregnant women with gestational diabetes mellitus (GDM) and comorbidities during Delta period. Schematic representation of GDM infections during COVID-19 Delta variant-of-concern (VOC) period along with comorbidities like diabetes, cardiovascular disease, hypertension, and asthma.

**Figure 2:**
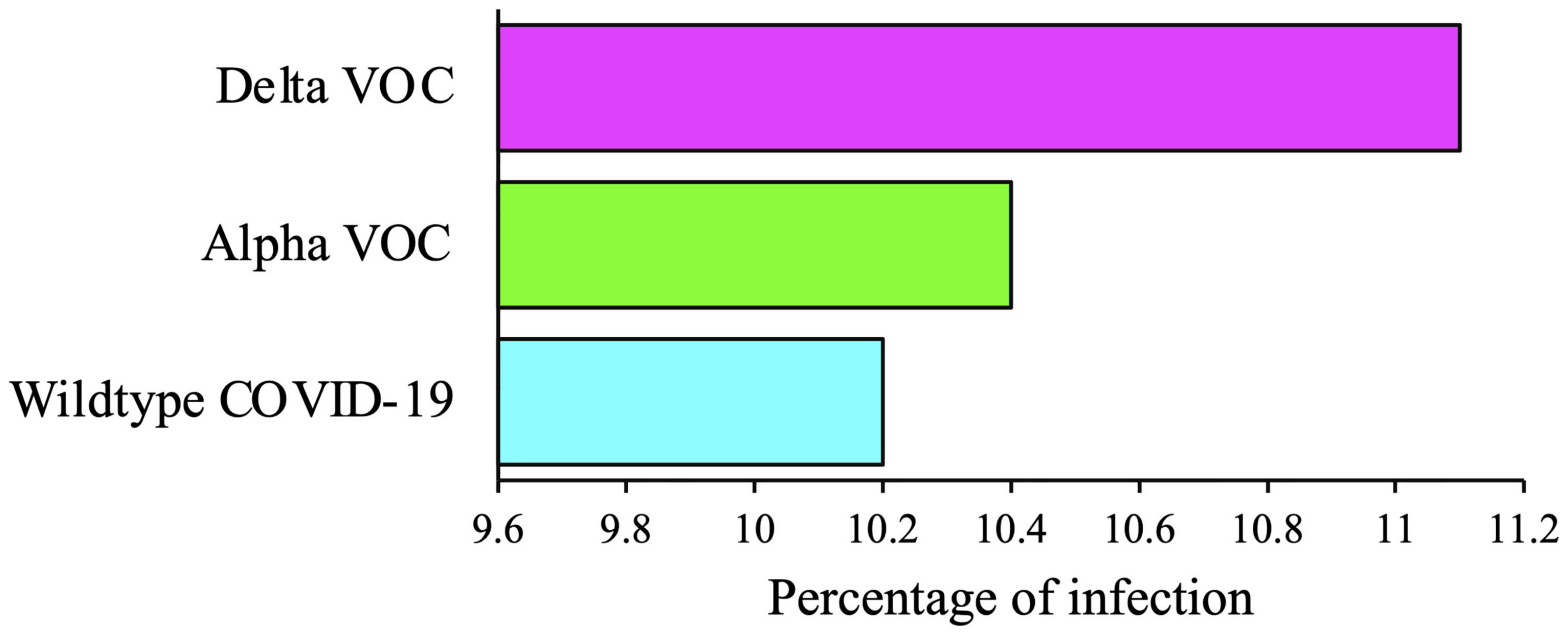
Gestational diabetes mellitus (GDM) with COVID-19 infections during wild-type, Alpha, and Delta periods. Comparison of COVID-19 infection rates in percentage among pregnant women with GDM during wild-type, Alpha VOC, and Delta VOC periods.

SARS-CoV-2 has continuously been evolving as a new variant since it was first identified in China through genetic mutations occurring during the replication of the genome. A variant, by definition, contains one or more mutations that separate it from other variants of the SARS-CoV-2 viruses. During the COVID-19 pandemic, different variants of SARS-CoV-2 have already been documented globally (11). Based on potential consequences of transmissibility, severity, and immunity of SARS-CoV-2 on epidemiological situation, variants were classified into i) variant-being-monitored (VBM), ii) variant-of-interest (VOI), iii) VOC, and iv) variant-of-high-consequence (VOHC) (12). The COVID-19 Delta VOC (B.1.617.2), a double mutated variant (E484Q and L452R) of wild-type SARS-CoV-2 (Wuhan strain), was first identified in India in December 2020 (13). In existing time, it is spreading throughout the globe at an alarming rate, faster than previous variants of SAR-CoV-2, and becoming the dominant strain in more than a dozen of countries; it is associated with high transmissibility (40%–60% more than Alpha variant and twice than Wuhan strain) and an increased risk of hospitalization (14, 15). The Delta variant is now responsible for more than 83% and 90% of COVID-19 cases being reported in the United States and the United Kingdom, respectively (15–17). In the European Union/European Economic Area (EU/EEA), in accordance with ECDC, 90% of new COVID-19 infections will be owing to the Delta variant by the end of August 2021 (16). Among the vulnerable groups of COVID-19 infections, pregnant women are more likely to get severe illness and symptoms of COVID-19 along with the possible requirement of admission to intensive care units since they are immunocompromised while Delta is highly contagious and infectious (18, 19). New surveillance by UK Obstetric Surveillance System reported an increase in the rate of hospitalization of pregnant women (54.2%) amid the spread of the Delta variant with severe illness than its previous waves (8). Although the association of disease severity with the Delta VOC infections during the period of pregnancy has not been well studied like wild-type COVID-19 strain and is until now under investigation, certain studies on the Delta-dominant period during pregnancy reported severe infections with worse pregnancy outcomes in contrast to the wild-type infections (8, 20). Pregnant women with preexisting medical complications like diabetes, hypertension, asthma, and cardiovascular diseases are more sensitive to COVID-19 infections, posing worse scenarios during pregnancy (8, 19, 20). Because GDM develops during pregnancy with an estimation of 1–28% depending on geographical locations, population characteristics, and diagnostics (10), it is imperative to know the possible risks and complications associated with COVID-19 infections, especially with the predominant Delta VOC to ensure maternal and neonatal sound health. A recent national and prospective observational cohort study in the United Kingdom consisting of 3,371 pregnant women revealed that infection severity increased during the Delta period (n = 171, 45%) compared with the wild-type (n = 1,435, 24.4%) and Alpha periods (n = 1,765, 35.8%) with increased risks of pneumonia (n = 171, 36.8%) and requiring more respiratory support (n = 171, 33.3%) and admission to intensive care units (n = 171, 15.2%). During the Delta period, the majority of pregnant people admitted to the hospital were aged ≥35 years (22.9%), and most of the pregnant women across all three periods were overweight or obese. The number of women admitted in the Alpha (n = 247, 14.0%) and Delta (n = 23, 13.5%) periods with one or more comorbidities was higher compared with that in the wild-type period (n = 169, 11.8%) (8). The study also showed that pregnant women having GDM are more vulnerable to the Delta VOC infection (n = 171, 11.1%) than wild-type variant (n = 1,435, 10.2%) and Alpha VOC (n = 1,765, 10.4%) (**Figure 1**). Additionally, GDM patients are among those groups that are most sensitive to Delta infections (n = 171, 11.1%), which is in contrast to pregnant people who have comorbidities like diabetes (n = 171, 1.2%), hypertension (n = 171, 2.9%), asthma (n = 171, 8.8%), and cardiovascular diseases (n = 171, 1.2%) (**Figure 2**) (8). During the Delta period, GDM patients are nine and three times more vulnerable to the Delta VOC infection than those with diabetes and cardiovascular diseases, and hypertension, respectively (**Figure 2**). A retrospective cohort study from India concluded that pregnant women with GDM during the Delta VOC dominant second wave had higher rates of infection than those in the first wave (20). Thus, Delta VOC provides an increased risk of infection to pregnancy, and pregnant mothers with GDM are more prone to Delta infections. However, more robust estimates of the maternal complications during pregnancy arising due to the Delta VOC infections are urgently needed to figure out health hazards associated with pregnancy.

## Management of Gestational Diabetes Mellitus During the Delta Variant-of-Concern Period

Pregnant women are more vulnerable to becoming severely ill from COVID-19 than non-pregnant women of a similar age in accordance with the WHO (17). Approximately 1 in 10 women admitted to the hospital with symptoms of COVID-19 require intensive care, and 1 in 5 pregnant women give birth prematurely (19). Since pregnant women with GDM have higher chances of infection during the Delta period, avoiding exposure to COVID-19 is the best way to prevent the disease. Vousden et al. conducted a study at the national level in the United Kingdom covering the wild-type, Alpha, and Delta periods and showed that over 99% of pregnant women admitted to the hospital with symptomatic COVID-19 were not unimmunized, which provides good news for pregnant women to be vaccinated against COVID-19 (8). Because the admissions of pregnant women to the hospital with COVID-19 are increasing and pregnant women appear to be more severely affected by the Delta variant of the disease, it is urgent to prioritize vaccination to pregnant women (8). Pregnant women with/without GDM or comorbidities should wear masks, practice good hand hygiene, and maintain social distance to protect pregnant themselves from infection (21). Importantly, wearing masks and maintaining physical distance have been shown to be effective to reduce the disease spread because the primary route of the viral transmission is through respiratory particles, and they transmit from both symptomatic and asymptomatic individuals. Thus, lowering disease spread requires limiting contacts of infected individuals *via* social distancing, and reducing the probability of transmission per contact *via* wearing masks (22). Pregnant women should regularly check blood glucose, avoid crowded places or mass gatherings, consult with clinicians regularly, and practice healthy life programming during the Delta period (23). Due to COVID-19 pandemic-controlling measures like lockdown, pregnant people with GDM or any other medical complications might have limited access to healthcare, medication, healthy diet, and healthy lifestyle; hence, self-care, in this situation, is the most prioritized measure to be adopted for these patients (24–28). During the COVID-19 pandemic, therapeutic approaches like telemedicine and digital care have been practiced globally, and these have become more effective, fruitful, promising, and successful in optimizing pregnancy care during the COVID-19 pandemic (23, 24, 26). Indeed, the tele-/digital medicine/care helps to avoid physical contact among those who are infected, uninfected, or clinicians in many situations like during quarantine or self-isolation or lockdown, thereby maintaining social distance to lower the spread of COVID-19. Although the remote monitoring of COVID-19 patients *via* telemedicine or other e-health technologies has improved the healthcare system, the deployment of e-devices and digital applications utilized to enhance screening and monitoring disease progression require accessibility for commoners and practitioners.

## Future Research Guidance

Although the Delta variant is spreading globally at a faster rate than any other previous variants of SARS-CoV-2, to our knowledge, until now there have been no national statistics or research surveillance studies conducted that address medical complications associated with Delta infections, especially in pregnant women with GDM. Since GDM patients have increased risks of pre-eclampsia, depression, and requirement of cesarean section, and the newborns are at an increased risk of becoming overweight and jaundice and developing type 2 diabetes, or even having a chance of stillbirth (29), it is urgent to conduct studies about pregnancy-associated complications following a Delta infection.

## Concluding Remarks

Delta-dominant period involves more severe infection and poor pregnancy outcomes than the other strains of SARS-CoV-2. In this scenario, a clear instructional piece of advice has been provided for the care of the mother and baby. Pregnant mothers, regardless of whether they have GDM or any preexisting medical complications, need to focus on self-care and be vaccinated against SARS-CoV-2 as early as possible. A policy should be made and implemented by prioritizing pregnant women under a quick vaccination program. Additional data are required from surveillances and cohort studies on SARS-CoV-2 infections during pregnancy for better understanding and patients’ counseling.

## Author Contributions

MM conceptually designed this study and wrote the manuscript. MK reviewed the overall manuscript by making critical comments and valuable inputs. All authors contributed to the article and approved the submitted version.

## Funding

MM and MK were supported by the initial postdoctoral fund of Henan Provincial People’s Hospital, Henan, China.

## Conflict of Interest

The authors declare that the research was conducted in the absence of any commercial or financial relationships that could be construed as a potential conflict of interest.

## Publisher’s Note

All claims expressed in this article are solely those of the authors and do not necessarily represent those of their affiliated organizations, or those of the publisher, the editors and the reviewers. Any product that may be evaluated in this article, or claim that may be made by its manufacturer, is not guaranteed or endorsed by the publisher.
